# Accommodating flourishing and depressive symptoms into the bifactor (S–1) model of mental health: longitudinal invariance and criterion validity

**DOI:** 10.3389/fpsyg.2026.1752388

**Published:** 2026-08-04

**Authors:** Chaoran Sun, Sylvia Kwok, Yu Huang

**Affiliations:** Department of Social and Behavioural Sciences, College of Liberal Arts and Social Sciences, City University of Hong Kong, Kowloon, Hong Kong SAR, China

**Keywords:** bifactor (S–1) model, criterion validity, depression, flourishing, longitudinal invariance, mental health

## Abstract

This study examined the longitudinal reliability and validity of the bifactor (S–1) model of mental health among 371 Chinese university students, refining the ﻿dual-factor model that views mental health as encompassing both flourishing and psychopathology. Using depression as the reference factor, participants completed measures of depressive symptoms (CES-D), flourishing (MHC-SF), and well-being (PERMA Profiler) at two time points. Factor analyses showed that the bifactor (S–1) model achieved excellent fit and interpretability, outperforming the symmetrical bifactor model by yielding fewer anomalous loadings and clearer separation of general and specific factors. Structural equation modeling further demonstrated that both the general reference factor and the flourishing-specific factor at baseline independently predicted multiple well-being domains at follow-up, controlling for demographics and baseline levels. These findings support the model’s criterion validity and temporal stability, confirming its effectiveness in capturing shared and unique aspects of mental health. Conceptually, the bifactor (S–1) approach provides evidence of the independence of flourishing ﻿with respect to depressive symptoms, ﻿meaning that people with mental illness are able to flourish despite their psychological issues.﻿ ﻿A practical implication is that ﻿notions such as “mental illness prevents people from﻿ attaining well-being﻿ may be invalid.” ﻿Many factors mitigate the impact of mental illness. With sufficient social support and psychoeducation, it is possible that people with psychological issues ﻿can thrive in society.

## 1 Introduction

According to the ﻿dual-factor model (Keyes, 2007), flourishing and depression are not ﻿simple opposites; the absence of symptoms does not necessarily mean mental flourishing﻿. ﻿This framework has guided intervention and research design [reviewed by Iasiello, 2023; Iasiello et al., 2020; Magalhães, 2024]. However, empirical evidence on the psychometric relationship remains mixed: some reviews report mostly bivariate ﻿relationships (Iasiello et al., 2020; Magalhães, 2024), whereas others identify evidence consistent with a bipolar or even one-factor structure (Schürmann-Vengels et al., 2023; Zhao and Tay, 2023). Overall, the literature suggests that treating ﻿the relationship between flourishing and depression as strictly bipolar or strictly bivariate may be too simplistic.

**Figure 1:**
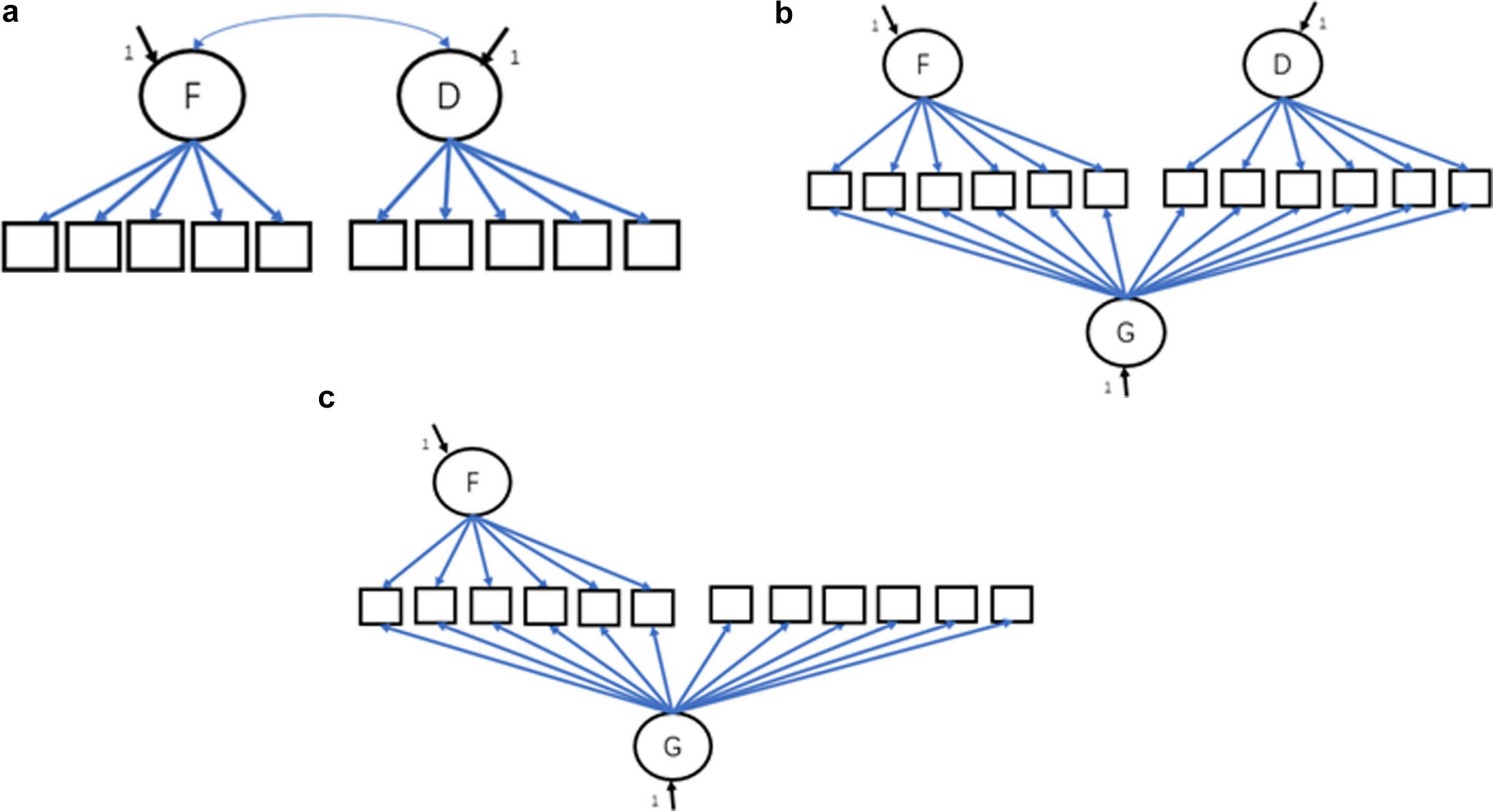
﻿﻿Correlated factors model, symmetrical bifactor model, and bifactor (S–1) model **﻿(a, b, c)**﻿. G, general factor; F, flourishing; D, depressive symptoms.

﻿The bipolar/bivariate nature of the relationship can be tested using the correlated-factors model (Figure 1a) to make the bipolar/bivariate inference (Keyes, 2007; Venning et al., 2013), which fits the observed variables ﻿onto the corresponding latent factors and ﻿correlates the two latent factors. As noted by Russell and Carroll (1999) and Tay et al. (2011), the correlation coefficient is the key evidence, with a negative correlation ﻿coefficient lower than −0.467 ﻿﻿indicates a bipolar or convergent relationship and ﻿one higher than −0.467 ﻿indicates a bivariate or divergent relationship. However, while the correlated-factors model itself does not ﻿presuppose a bipolar ﻿orbivariate ﻿relationship, it ﻿is easily misused in literature. In previous ﻿studies such as Black et al.’s (2019) study, the correlation ﻿coefficient between internalizing symptoms and well-being concepts was −0.58. Because this correlation falls below the widely accepted threshold of |0.85|, the authors concluded that the constructs exhibit discriminant validity (divergence) and ultimately favored a correlated-factors specification over a traditional symmetrical bifactor model, which suffered from structural anomalies like a vanishing specific factor. Nonetheless, from a convergent perspective, a latent correlation of |*r*| = 0.58 indicates a substantial degree of shared variance. In psychometric modeling, when factor correlations exceed moderate thresholds (e.g., >|0.467|), they signal a strong underlying convergent relationship that a correlated-factors model cannot explicitly partition (Russell and Carroll, 1999). A bifactor model is conceptually better suited to capture this shared variance via an overarching general factor while simultaneously preserving construct specificity. To leverage the advantages of the bifactor approach while bypassing the vanishing factor anomalies highlighted by Black et al. (2019), the present study utilizes a bifactor (S–1) model. This framework allows us to directly model the strong convergent relationship with depression without encountering the structural instability typical of traditional symmetrical bifactor configurations. This dual-level representation allows the bifactor model to align more closely with emerging psychometric evidence suggesting bipolarity in the relationship between flourishing and depression (Zhao and Tay, 2023).

In the present study, the symmetrical bifactor model (Figure 1b) is therefore included as a natural benchmark for testing the presence of a general mental health factor which accommodates the shared variance between flourishing and depression. The comparison with alternative models is ﻿performed to evaluate both model fit and the interpretability of the general and specific factors. The symmetrical bifactor model structure, which includes a general factor loading ﻿onto all observed variables and specific factors loading ﻿onto their corresponding subsets (Reise et al., 2010; Reise et al., 2007; Rodriguez et al., 2016b), ﻿offers a perspective on accommodating neither ﻿a same-continuum﻿nor ﻿an independent relationship between the ﻿two concepts. The bifactor model structure allows for the assessment of a unidimensional structure between flourishing and depression while accounting for unique variance attributable to specific constructs once shared variance is extracted (Reise, 2012; Reise et al., 2010; Reise et al., 2007). Hides et al. (2020) compared five model structures, including the bifactor model and the correlated-factors model, and found the bifactor model provided the best model fit. However, the symmetrical bifactor model has limitations. Specifically, loading all items ﻿onto both the general and specific factors can result in anomalous factor loadings usually on the specific factor (Eid et al., 2017). ﻿These anomalous factor loadings are weak loading issues that bring ﻿about model validity and interpretability concerns. Given that model interpretability is ﻿thus also a critical concern﻿, in addition to﻿ model fit, the bifactor (S–1) model ﻿has been introduced (Figure 1c), in which one of the specific factors is omitted to anchor the general factor, thereby stabilizing the factor loadings of the remaining general structure and maintaining interpretability.

A critical consideration in the bifactor (S–1) model is the selection of﻿ the reference facet. Black et al. (2019), for instance, applied this model to internalizing and externalizing symptoms along with well-being. They found that using internalizing symptoms as the reference facet resulted in better model fit and fewer anomalous loadings compared ﻿﻿with the symmetrical bifactor model. In contrast, using well-being as the reference facet led to model misspecification. Given that the Mental Health Continuum (Keyes, 2002) was developed with reference to the diagnostic criteria for clinical depression, and in light of Black et al.’s (2019) findings, depression appears to be a theoretically and empirically justified choice for the reference facet in a bifactor (S–1) model of mental health. Nonetheless, since Black et al. (2019) used cross-sectional data, conclusions about longitudinal invariance and criterion validity remain unaddressed.

Psychometric research has increasingly conceptualized depression as a multidimensional construct rather than strictly unidimensional, comprising affective, cognitive, and somatic symptom clusters that exhibit both shared and specific variance (Balsamo et al., 2013). Studies employing factor-analytic and item response theory approaches have demonstrated that depressive symptoms, particularly in self-report instruments, tend to reflect correlated but distinct dimensions, such as pessimism and self-criticism, rather than a single homogeneous factor (Balsamo et al., 2013; Kendler et al., 2011). Recognizing this multidimensionality is critical when designating depression as the reference factor in the bifactor (S–1) model, as some symptom dimensions may share variance with flourishing while others remain distinct. The present study aims to contribute to the ﻿dual-factor model literature by providing longitudinal evidence on the validity and reliability of the bifactor (S–1) model with flourishing as the specific factor and depressive symptoms as the general reference factor. It is critical to clarify that the bifactor (S–1) model does not take a ﻿stance on ﻿the bipolar ﻿versus bivariate hypotheses; instead, it *addresses* this debate empirically by modeling the shared variance between depressive symptoms and flourishing so their relationship ﻿can be understood as related but distinct rather than bipolar or bivariate. Additionally, bifactor models nearly always fit better than their correlated factor siblings (Gignac, 2016; Rodriguez et al., 2016a), which ﻿necessitates additional model parameter examinations beyond omnibus model fit necessary. Therefore, we examined explained common variance (ECV)﻿ and the omega statistics, (*ω*, and ω_h_﻿) to assess the strength of the general factor and the interpretability of the bifactor model, rather than relying on omnibus model fit indices alone. ﻿We also examined the correlation between the symmetrical and bifactor (S–1) ﻿models to reveal the stability of the symmetrical bifactor model factors. If the correlation is exceptionally ﻿strong (e.g., |*r*| > 0.80), it﻿ would ﻿indicate that the symmetrical model’s factor is stable and untainted by anomalous loading shifts. ﻿A weaker correlation would empirically ﻿indicate that the symmetrical model has suffered from an anomalous loading issue, shifting the conceptual meaning of its factors (Eid et al., 2018; Heinrich et al., 2023).

## 2 Methods

### 2.1 Sample

﻿The participants in this study were college students in Mainland China recruited via two-wave convenience sampling. Ethical approval was obtained from the affiliated institution of the authors. To maximize representativeness within practical constraints, participants were recruited from a wide range of universities and regions across Mainland China using the Credamo Data Collection Platform.^1^ Credamo enables access to a diverse pool of respondents, which is widely used by university students in both urban and rural areas. Because Internet-based data collection and the paper-and-pencil approach are generally equivalent in terms of data collection procedures (Weigold et al., 2013), the questionnaire was designed online via Credamo and distributed to participants with financial compensation. To ﻿filter out bot ﻿responses, we utilized several attention check items which were rated as moderately effective (Storozuk et al., 2020; Xu et al., 2022). Given the longitudinal design’s anticipated 50% attrition rate (Hedeker et al., 1999), we employed an enriched recruitment strategy with two baseline data collections to ensure adequate sample size for subsequent analyses. Our ﻿required sample size estimate as higher than﻿ of > 300 was ﻿inferred from the sample size comparisons ﻿with previous scholars (Wolf et al., 2013). The initial baseline data collection took place in December 2023, and 500 surveys were returned. We collected 300 more baseline surveys in January 2024 to ensure a sufficient number of candidates for the second wave. The second wave was collected in May 2024 and June 2024, sending to the respondents of Wave 1. The chosen time interval was primarily for testing model stability. The ﻿participating ﻿university students ﻿were recruited via online social media (﻿e.g., WeChat, QQ, email, etc.). ﻿The participants were compensated with 10 RMB/wave to complete the survey. They provided informed consent by clicking the “agree” button on the online consent form before starting the survey. ﻿﻿Respondents aged younger than 18 years were excluded. The data﻿ from this current study ﻿are available upon request from the corresponding author.

### 2.2 Measurements

#### 2.2.1 Flourishing

The 14-item ﻿Mental Health Continuum-Short Form (MHC-SF) (Lamers et al., 2011) was adopted to measure flourishing. This instrument assesses flourishing from emotional, psychological, and social flourishing. ﻿An example item is “In the past month, how often do you feel happy?” Participants indicated their response on a 6-point Likert scale from 0 (“Never”) to 5 (“Everyday”). This scale has been translated into Chinese and has demonstrated good reliability (Guo C. et al., 2015). The total summed score represents the overall flourishing score. The reliability of the scale in the current study was good, with Cronbach’s *α*_T1_ = 0.94; Cronbach’s *α*_T2_ = 0.94.

#### 2.2.2 Depressive symptoms

The ﻿Center for Epidemiological Studies-Depression instrument CES-D﻿, (Radloff, 1977) was used to measure symptoms associated with depression. It includes 20 items assessing the frequency of depressive symptoms experienced in the past week. ﻿A sample item from the CES-D is: “I felt that everything I did was an effort.” Participants respond to each item on a 4-point scale, ranging from 0 (“Rarely or none of the time”) to 3 (“Most or all of the time”), providing a possible range of total scores from 0 to 60, with higher scores indicating greater depressive symptoms. The scale has been translated into Chinese and has shown satisfactory reliability and validity (Zhang et al., 2010). ﻿Items ﻿8, ﻿12, and ﻿16 ﻿are reverse coded. The scale’s reliability in the current study was good, with Cronbach’s *α*_T1_ = 0.94; Cronbach’s *α*_T2_ = 0.95.

#### 2.2.3 PERMA

The PERMA Profiler developed by Butler and Kern (2016) was used in the current study for criterion validity. It is a multidimensional instrument developed to assess well-being based on the five pillars outlined by Seligman: Positive Emotion, Engagement, Relationships, Meaning, and Accomplishment. Participants respond to the items based on a 10-point Likert scale. The response anchors are 0 (“Never”) to 10 (“Always”) and 0 (“Not at all”) to 10 (“Completely”) depending on the item content. The scale has been translated into Chinese and has been proved to be reliable and valid (Nie et al., 2024; Qian et al., 2025). Each dimension ﻿can be scored, ﻿separately, using the average of the three corresponding items. ﻿However, in the current study used the ﻿scores of each single dimension of PERMA ﻿were used separately as observed variables and did not use the total summed score﻿ was not used.

### 2.3 Statistical analysis

Descriptive data analysis and data cleaning were performed using SPSS version 26 (IBM Corp, 2019). To explore ﻿the underlying structure, we conducted a series of exploratory factor analysis (EFA), exploratory bifactor analysis (EBFA), and confirmatory factor analysis (CFA) via MPlus 8.3 (Muthén and Muthén, 2019) and R 4.4.2 (R Core Development Team, 2024). EFA and EBFA were conducted on the attrition data. CFA was conducted on the longitudinal data. The skewness and kurtosis of the observed variables were calculated to examine whether the data fit the ﻿assumptions﻿ of structural equation modeling (SEM). The criteria for data normality were defined as absolute skewness ≤2.0 and absolute kurtosis ≤7.0 (Curran et al., 1996; Kline, 2023). The ﻿method of Maximum Likelihood with Mean and Variance Adjusted for Missing Data under Missing at Random (MLM) method was used for estimation, consistent with the distribution pattern of the data. ﻿Factor loadings on the specific factor lower than |0.30| would be deemed as weak and indicate model validity issue (Howard, 2016). The MPlus and R codes ﻿are available at https://osf.io/jtxh4.

When evaluating a multidimensional psychological scale, researchers must decide whether to report a single, overarching total score or individual subscale scores. A bifactor model offers an elegant statistical framework to test this by specifying that the variance of each item is explained simultaneously by two sources:

1. A single general factor that reflects the common construct underlying all scale items.

2. Multiple specific factor(s) that capture the unique, residual variance shared among subscale items after controlling for the general factor.

While traditional internal consistency metrics like Cronbach’s alpha can overestimate reliability in multidimensional data, ﻿the coefficient ﻿omega provides a more accurate breakdown:

- Omega total ( ω ): Represents the total reliable variance explained by *both* the general factor and the specific subscale factors combined.
- Omega hierarchical ( $\omega_{h}$ ): Isolates and represents *only* the proportion of systematic variance accounted for by the overarching general factor.
- ECV: While metrics like omega hierarchical ( $\omega_{h}$ ) ﻿quantify a construct’s reliability relative to all variance (including random measurement error), ECV focuses strictly on the common (shared) factor variance. It ﻿is calculated as the ratio of variance explained by the ﻿general factor relative to the total variance explained by all factors (general factor + all specific factors) combined.

In practice: ﻿comparing these two values reveals whether subscales add unique value. If $\omega_{h}$ is exceptionally high (e.g., >0.80) and very close to ω , it means that the vast majority of the scale’s reliable variance is driven by the general factor. In such cases, the subscales possess very little unique variance, and reporting distinct subscale scores in practice may be redundant or misleading. ECV provides a direct measure of “factor dominance.” It answers the question: ﻿“Of the structure ﻿that has been successfully modeled, how much is driven by the main trait versus the subscales? ﻿A higher general-factor ECV (e.g., > 0.60 or 0.70)﻿﻿indicates a strong, dominant general factor.

#### 2.3.1 Exploratory analysis on the attrition data

We ﻿performed EFA on the attrition data by successively extracting two to three latent factors using varimax rotation. In the initial baseline EFA, a varimax (orthogonal) rotation was intentionally applied to mimic the uncorrelated, independent factor structure characteristic of a classic bifactor model. Recognizing that this orthogonal constraint routinely yields a poorer fit when applied to naturally overlapping constructs, we subsequently executed an EFA using a promax (oblique) rotation to let the primary dimensions freely correlate. This step served as the necessary foundation for the EBFA, wherein we applied the Schmid-Leiman (S-L) transformation. The S-L orthogonalization procedure explicitly assumes an underlying hierarchical or bifactor topology, transforming the correlated promax primary factors into a bi-level structure containing a single, orthogonal general factor and distinct, group-specific factors. The ﻿CFAs were later conducted using data from the longitudinal sample, ﻿rather than the attrition data.

#### 2.3.2 Confirmatory analyses on the second wave data

The CFA model results were compared between the symmetrical and bifactor (S–1) ﻿models to determine which model should be adopted regarding their model fit and factor loading results. The appropriateness of the model was assessed based on model fit indices including CFI, TLI, SRMR, and RMSEA. The cutoff values of CFI and TLI ﻿are as follows: above 0.90 ﻿indicates an acceptable fit, ﻿and values above 0.95 suggesting an excellent fit (Hu and Bentler, 1999). ﻿For SRMR ﻿values below 0.08 indicating a good fit (Hu and Bentler, 1999). ﻿For RMSEA﻿, values cutoff was below 0.08,﻿ indicate an acceptable fit, ﻿and values below 0.05, ﻿indicate a close fit (Browne and Cudeck, 1992).

H index, omega statistics, ﻿ECV﻿, omega hierarchical (Omega H), factor determinacy, and ﻿percentage of uncontaminated correlation (PUC) indices were calculated to determine the appropriateness ﻿of adopting a symmetrical or S–1 model structure at the confirmatory stage. H indices higher than 0.70 ﻿are deemed as acceptable (Hancock and Mueller, 2001). Omega﻿ values higher than 0.70 indicates strong internal consistency (McDonald, 1999). ﻿An ECV higher than 0.70 indicates one-dimensionality and ﻿an ECV lower than 0.70 indicates multi-dimensionality (Liu et al., 2023; Reise et al., 2010). ﻿ω_h_﻿ quantifies the proportion of variance in total scores attributable to a factor and a value >0.50 is often considered ﻿the minimum ﻿threshold for a strong factor (Reise et al., 2010; Reise et al., 2013). Factor determinacy measures how well a latent factor can be precisely recovered from its indicators in SEM (McDonald and Mulaik, 1979). Values range from 0 (no precision) to 1 (perfect); higher ﻿values﻿ indicate more reliable factor scores with low error variance. PUC reflects the proportion of item correlations explained by the general factor in a bifactor model and a value >0.80 is considered an indicator of uni-dimensionality (Reise et al., 2013).

#### 2.3.3 Invariance analyses on the two-wave data

Configural, metric, and scalar invariance were tested to ensure that the bifactor structure was equivalent across the time points﻿, using MPlus 8.3 (Muthén and Muthén, 2019). To test whether the fit of the model was equivalent across time, we relied on statistical differences in the CFI, RMSEA, and Akaike Information Criteria (AIC) between the constrained and unconstrained models (Putnick and Bornstein, 2016). If the constrained and unconstrained models were statistically variant and the changes in model fit indices exceeded the following criteria, the null hypothesis of invariance would be rejected: ΔCFI > 0.010 and ΔRMSEA > 0.015 (Kline, 2023), and ΔAIC > 20 (Burnham and Anderson, 2002).

#### 2.3.4 Model comparisons

﻿Factor scores estimated using R 4.4.2 (R Core Development Team, 2024) were plotted. The general factor from the symmetrical bifactor model was correlated with the general reference factor from the bifactor (S–1) model. The flourishing specific factors from both models were correlated. Since the general reference factor was anchored by the depression items, the general reference factor from the bifactor (S–1) model was correlated with the depression ﻿- specific factor from the symmetrical bifactor model to explore their relationship.

#### 2.3.5 Criterion validity

The R package lavaan (Rosseel, 2012) was used in R 4.4.2 (R Core Development Team, 2024) to model the predictive relationship between the bifactor (S–1) model at T1 and the criterion variables at T2, adjusting for age, gender, and familial income. The five pillars from the PERMA-Profiler (Butler and Kern, 2016): positive affect, engagement, relationship, meaning, and achievement were used as the criterion variables. The variable scores at T1 were also included in the SEM model to control for baseline effect. The appropriateness of model specification was assessed based on model fit indices including CFI, TLI, SRMR, and RMSEA.

## 3 Results

The demographic details of the longitudinal and attrition ﻿samples are presented in Table 1. ﻿﻿The assumption test ﻿for skewness and kurtosis﻿ on the longitudinal sample are in Supplementary Table S1. There were 784 valid responses ﻿in Wave 1 after removing the duplicates. There were 372 participants ﻿who returned for the second wave and 371 participants retained for SEM after data cleaning. ﻿Female participants comprised 68.5% ﻿of those in the longitudinal data. In the longitudinal sample, 3% were freshmen, 15.6% were ﻿sophomores, 30.5% were junior, 30.7% were senior, and the rest of them were either in extension of study or in graduate school. ﻿Majorities of the participants ﻿were from married families, ﻿were of Han ethnicity, ﻿﻿had no or one sibling, and ﻿had never received any psychiatric diagnosis.

**Table 1:** Demographic data of the longitudinal (*N* = 371) and attrition sample (*N* = 412).

Variable	*n* (%) (*N* = 371)	*n* (%) (*N* = 412)
Sociodemographic characteristics		
Sex		
Female	254 (68.5%)	264 (64.1%)
Male	117 (31.5%)	148 (35.9%)
Age		
18	7 (1.9%)	16 (3.9%)
19	20 (5.4%)	24 (5.8%)
20	57 (15.4%)	54 (13.1%)
21	81 (21.8%)	75 (18.2%)
22	65 (17.5%)	60 (14.6%)
23	44 (11.9%)	53 (12.9%)
24	28 (7.5%)	35 (8.5%)
Older than 24	69 (18.6%)	95 (23.1%)
Ethnicity		
Han	356 (96%)	393 (95.4%)
Ethnic minority	15 (4.0%)	19 (4.6%)
No. siblings		
0	103 (27.8%)	150 (36.4%)
1	178 (48%)	149 (36.2%)
2	73 (19.7%)	88 (21.4%)
3 or more	17 (4.6%)	25 (6.1%)
Parent’s educational level		
Middle school or less	117 (31.5%)	118 (28.6%)
High school graduate or unfinished	83 (22.4%)	85 (20.6%)
Professional high school	23 (6.2%)	21 (5.1%)
Professional college	63 (17%)	55 (13.3%)
Bachelor’s	75 (20.2%)	111 (26.9%)
Master’s	7 (1.9%)	18 (4.4%)
Doctorate	3 (0.8%)	4 (1.0%)
Parent’s marital status		
Married	346 (93.3%)	375 (91.0%)
Divorced	19 (5.1%)	28 (6.8%)
Widowed	4 (1.1%)	6 (1.5%)
Other	2 (0.5%)	3 (0.7%)
Yearly household income		
Less than 100 k CNY	101 (27.2%)	111 (26.9%)
100 k to 200 k CNY	172 (46.4%)	192 (46.6%)
200 k to 300 k CNY	72 (19.4%)	63 (15.3%)
300 k to 400 k CNY	20 (5.4%)	28 (6.8%)
More than 400 k CNY	6 (1.6%)	18 (4.4%)
Previous received psychiatric diagnosis		
No	338 (91.1%)	369 (89.6%)
Yes	33 (8.9%)	43 (10.4%)

### 3.1 Attrition analysis

Those who returned for the second wave (*n* = 372) before data cleaning were not significantly different from those who dropped out (*n* = 412) ﻿in terms of gender [ $\chi^{2}(1)=1.54,p>.05$ ], ethnicity [ $\chi^{2}(2)=3.47,p>.05$ ], age group [ $\chi^{2}(8)=11.08,p>.05$ ], parental marriage status [ $\chi^{2}(4)=4.68,p>.05$ ], annual family income [ $\chi^{2}(5)=10.92,p>.05$ ], parental education [ $\chi^{2}(7)=14.01,p>.05$ ], previous psychiatric diagnosis, flourishing [*t*(782) = 1.48, *p* >.05] , depressive symptoms [*t*(782) = 0.61, *p* >.05 ], positive affect﻿ (PERMA-Profiler) [*t*(782) = 1.33, *p* >.05 ], relationship ﻿(PERMA-Profiler) [*t*(782) = −0.17, *p* >.05 ], ﻿or meaning﻿ (PERMA-Profiler) [*t*(782) = 1.01, *p* >.05 ]. Those who returned for the second wave had higher average for engagement﻿ (PERMA-Profiler) [*t*(782) = 2.64, *p* = 0.004] and achievement﻿ (PERMA-Profiler) [*t*(782) = 1.68, p=.05 ] constructs, and had more siblings [ $\chi^{2}(4)=15.33,p=.004$ ]. This might potentially enhance﻿ the need for interpretive caution.

### 3.2 Model results

To determine the optimal underlying dimensionality of the scale (Table 2), an EFA was conducted on the attrition sample. A two-factor model was systematically compared against an extracted three-factor solution to evaluate structural clarity and statistical fit. The factor loadings and item communalities (*h*^2^) for both solutions are presented in Table 2. The three-factor model was retained as the superior psychometric framework based on several empirical indicators: transitioning from the two-factor to the three-factor model yielded a widespread increase in item communalities, indicating that a greater proportion of item variance was captured by the latent structure. Notable variance gains were observed across both flourishing and depression items, including﻿ the flourishing indicators F1 (increasing from 0.48 to 0.53), F2 (0.52 to 0.58), and F3 (0.62 to 0.68), as well as﻿ the symptom indicators D6 (0.62 to 0.65), D15 (0.51 to 0.55), and D19 (0.41 to 0.49). The flourishing items F1, F2, F3, and F4 ﻿loaded heavily ﻿onto both factor 3 and factor 1 and the depression block: D1, D5, D6, D8, D12, D16, D18 ﻿loaded significantly ﻿onto all three factors simultaneously. For this reason, a three-factor model structure was preliminarily accepted, ﻿in addition to the better model fit results [ $\chi^{2}(462)\;$ = 982.80, *p* < 0.001, RMSEA = 0.05, SRMR = 0.03] ﻿compared with the two-factor model [ $\chi^{2}(494)$ = 1213.48, *p* < 0.001, RMSEA = 0.06, SRMR = 0.04]﻿, a three-factor model structure was preliminarily accepted. The three-factor solution was selected not only due to comparatively better fit, but also because it provided a more interpretable structure and a suitable foundation for EBFA and bifactor modeling. EBFA on the attrition data ﻿revealed three misaligned items (﻿Items 8, ﻿12, and ﻿16) in the depression specific factor for the symmetrical bifactor model (Table 3). These three items are reverse-coded questions which are likely to overlap with the flourishing specific factor (factor loadings on the flourishing specific factor: *λ*_8_ = − 0.24; *λ*_12_ = − 0.24; *λ*_16_ = −0.28). Due to ﻿their reverse-﻿coded nature, these three items were dropped from further analysis.

**Table 2:** Path estimates for the two to four-factor models in EFA after Varimax rotation using the attrition data (*N* = 412).

	Two-factor			Three-factor			
Items	1	2	*h* ^2^	1	2	3	*h* ^2^
F1	0.59	−0.36	0.48	0.47	−0.26	−0.49	0.53
F2	0.60	−0.39	0.52	0.47	−0.28	−0.53	0.58
F3	0.68	−0.40	0.62	0.54	−0.28	−0.56	0.68
F4	0.74	−0.21	0.60	0.65	−0.14	−0.41	0.61
F5	0.62	−0.37	0.52	0.60	−0.36	−0.20	0.53
F6	0.69	−0.34	0.59	0.67	−0.33	−0.22	0.60
F7	0.74	−0.26	0.62	0.70	−0.23	−0.26	0.62
F8	0.67	−0.27	0.51	0.69	−0.28	−0.10	0.56
F9	0.65	−0.34	0.54	0.65	−0.34	−0.17	0.57
F10	0.55	−0.38	0.45	0.53	−0.37	−0.19	0.46
F11	0.64	−0.30	0.49	0.66	−0.32	−0.08	0.55
F12	0.73	−0.23	0.59	0.72	−0.22	−0.19	0.60
F13	0.75	−0.16	0.59	0.73	−0.15	−0.21	0.59
F14	0.77	−0.26	0.66	0.72	−0.23	−0.31	0.66
D1	−0.48	0.56	0.54	−0.36	0.47	0.46	0.56
D2	−0.28	0.46	0.29	−0.27	0.46	0.13	0.30
D3	−0.31	0.61	0.46	−0.24	0.55	0.31	0.46
D4	−0.42	0.43	0.36	−0.37	0.39	0.26	0.36
D5	−0.38	0.60	0.51	−0.32	0.55	0.32	0.51
D6	−0.44	0.66	0.62	−0.32	0.57	0.47	0.65
D7	−0.33	0.70	0.60	−0.24	0.62	0.41	0.61
D8	−0.55	0.48	0.54	−0.47	0.42	0.40	0.55
D9	−0.37	0.69	0.61	−0.31	0.63	0.34	0.61
D10	−0.19	0.69	0.51	−0.14	0.65	0.24	0.51
D11	−0.26	0.44	0.26	−0.21	0.40	0.23	0.26
D12	−0.58	0.54	0.62	−0.47	0.46	0.46	0.64
D13	−0.29	0.50	0.34	−0.27	0.49	0.17	0.34
D14	−0.39	0.62	0.54	−0.39	0.63	0.16	0.57
D15	−0.20	0.69	0.51	−0.20	0.70	0.11	0.55
D16	−0.60	0.46	0.57	−0.51	0.38	0.43	0.59
D17	−0.12	0.46	0.23	−0.13	0.48	0.06	0.25
D18	−0.47	0.60	0.58	−0.36	0.51	0.46	0.60
D19	−0.19	0.62	0.41	−0.22	0.66	−0.001	0.49
D20	−0.20	0.68	0.51	−0.18	0.66	0.20	0.51

F1–F14, flourishing indicators; D1–D20, depression indicators.

**Table 3:** Schmid Leiman absolute values of factor loadings (>|0.2|) on the attrition sample (*N* = 412).

T1	*g*	F1*	F2*	*h* ^2^
F1	0.62	0.30		0.48
F2	0.65	0.30		0.52
F3	0.7	0.35		0.62
F4	0.62	0.45		0.60
F5	0.64	0.32		0.52
F6	0.67	0.38		0.59
F7	0.65	0.44		0.62
F8	0.61	0.38		0.51
F9	0.65	0.35		0.54
F10	0.6	0.26		0.45
F11	0.61	0.35		0.49
F12	0.62	0.44		0.59
F13	0.59	0.47		0.59
F14	0.67	0.45		0.66
D1	−0.67		0.24	0.54
D2	−0.48		0.24	0.29
D3	−0.59		0.33	0.46
D4	−0.55			0.36
D5	−0.64		0.30	0.51
D6	−0.71		0.33	0.62
D7	−0.67		0.38	0.60
D8	−0.68	−0.24		0.54
D9	−0.69		0.36	0.61
D10	−0.57		0.42	0.51
D11	−0.46		0.23	0.26
D12	−0.72	−0.24	0.20	0.62
D13	−0.52		0.26	0.34
D14	−0.66		0.32	0.54
D15	−0.58		0.42	0.51
D16	−0.69	−0.28		0.57
D17	−0.38		0.29	0.23
D18	−0.69		0.27	0.58
D19	−0.52		0.37	0.41
D20	−0.57		0.41	0.51

F1–F14, flourishing indicators; D1–D20, depression indicators.

The standardized factor loadings of the symmetrical and bifactor (S–1) ﻿models were compared using the second wave data from the longitudinal sample (Tables 4, 5). The model fit indices of the symmetrical bifactor model ﻿were $\chi^{2}(403)$ = 635.39, *p* < 0.001, CFI = 0.94, TLI = 0.93, RMSEA = 0.05, SRMR = 0.04. ﻿Those of the bifactor (S–1) model are $\chi^{2}(420)$ = 720.53, *p* < 0.001, CFI = 0.94, TLI = 0.93, RMSEA = 0.05, SRMR = 0.04. The symmetrical bifactor model results revealed anomalous factor loadings on the flourishing specific factor according to Howard’s (2016) recommended criteria. As shown in Table 6, compared ﻿with the symmetrical model, the bifactor (S–1) model ﻿had acceptable H ﻿index, omega, and factor determinacy ﻿values for the latent factors, which means that the factors were well-defined and reliable. The ﻿ω_h_ was below ﻿the threshold for the general reference factor. The low ﻿ω_h_ values primarily caution against interpreting depressive symptoms and flourishing total scores as pure indicators of the general reference factor but do not indicate that the general or flourishing specific factors are weak across the bifactor models. The ECV﻿ value shows that the general reference factor explained most ﻿of the common variance (0.80) while the flourishing specific factor still explained ﻿a substantial ﻿amount of common variance (0.42). The PUC of the bifactor (S–1) ﻿model’s general reference factor was strong at the level of 0.80, ﻿indicating low bias in﻿ the unidimensional treatment (Table 6).

**Table 4:** Standardized path estimates of the symmetrical bifactor model (*N* = 371).

Factors	Path estimates	SE
General		
F1	0.75	0.04
F2	0.82	0.02
F3	0.83	0.02
F4	0.70	0.03
F5	0.70	0.03
F6	0.72	0.03
F7	0.73	0.03
F8	0.65	0.04
F9	0.71	0.03
F10	0.61	0.03
F11	0.69	0.03
F12	0.72	0.03
F13	0.70	0.03
F14	0.78	0.02
D1	−0.66	0.03
D2	−0.44	0.05
D3	−0.64	0.03
D4	−0.59	0.04
D5	−0.59	0.04
D6	−0.64	0.03
D7	−0.64	0.03
D9	−0.64	0.03
D10	−0.44	0.05
D11	−0.43	0.05
D13	−0.42	0.05
D14	−0.60	0.04
D15	−0.46	0.04
D17	−0.37	0.05
D18	−0.61	0.04
D19	−0.48	0.04
D20	−0.52	0.04
Flourishing		
F1	0.26	0.07
F2	0.10^ns^	0.07
F3	0.04^ns^	0.06
F4	−0.21	0.08
F5	−0.19	0.07
F6	−0.32	0.07
F7	−0.34	0.06
F8	−0.29	0.07
F9	−0.08 ^ns^	0.07
F10	−0.16	0.06
F11	0.001 ^ns^	0.06
F12	−0.20	0.07
F13	−0.18	0.07
F14	−0.20	0.07
Depression		
D1	0.34	0.04
D2	0.42	0.04
D3	0.48	0.04
D4	0.26	0.04
D5	0.43	0.04
D6	0.45	0.04
D7	0.50	0.03
D9	0.46	0.04
D10	0.51	0.04
D11	0.31	0.05
D13	0.49	0.04
D14	0.42	0.04
D15	0.46	0.04
D17	0.48	0.04
D18	0.48	0.04
D19	0.49	0.04
D20	0.51	0.04

^ns^, non-significant. Other than the estimates which were marked as ^ns^; the others were significant at *p* < 0.01 level.

**Table 5:** Standardized path estimates of the bifactor (S–1) model (*N* = 371).

General	Path estimates	SE
F1	−0.63	0.04
F2	−0.64	0.03
F3	−0.66	0.03
F4	−0.47	0.04
F5	−0.57	0.04
F6	−0.55	0.04
F7	−0.57	0.04
F8	−0.48	0.05
F9	−0.61	0.04
F10	−0.51	0.04
F11	−0.55	0.04
F12	−0.51	0.04
F13	−0.56	0.04
F14	−0.57	0.04
D1	0.74	0.02
D2	0.60	0.04
D3	0.80	0.02
D4	0.63	0.05
D5	0.73	0.03
D6	0.79	0.02
D7	0.81	0.02
D9	0.79	0.02
D10	0.65	0.04
D11	0.53	0.04
D13	0.63	0.04
D14	0.73	0.03
D15	0.64	0.04
D17	0.57	0.05
D18	0.78	0.02
D19	0.67	0.04
D20	0.73	0.03
Flourishing		
F1	0.30	0.04
F2	0.45	0.03
F3	0.45	0.03
F4	0.58	0.04
F5	0.45	0.04
F6	0.53	0.04
F7	0.52	0.04
F8	0.50	0.04
F9	0.39	0.04
F10	0.39	0.04
F11	0.41	0.04
F12	0.58	0.04
F13	0.45	0.04
F14	0.58	0.04

All the estimates were significant at *p* < 0.001 level.

**Table 6:** Psychometric indices for each latent factor defined in symmetrical bifactor and bifactor (S–1) models specifying two/one specific factor(s) on the second wave data (*N* = 371).

Factors	General	Flourishing	Depression
Index	Symmetrical bifactor model		
*H*	0.96	0.38	0.81
ECV	0.75	0.07	0.41
ω	0.81	0.95	0.94
ω_h_	0.02	0.03	0.39
Factor determinacy	0.97	0.73	0.90
PUC	0.52		
	Bifactor (S–1) model		
*H*	0.96	0.81	
ECV	0.80	0.42	
ω	0.80	0.94	
ω_h_	0.21	0.39	
Factor determinacy	0.98	0.91	
PUC	0.80		

H, index of construct replicability; ECV, explained common variance due to the factor itself; PUC, percent correlations that are uncontaminated; ω_h_, Omega-hierarchical.

The general factor from the symmetrical bifactor model ﻿had a satisfactory H ﻿index (*H* = 0.96), but the flourishing specific factor’s H was low (H = 0.38), ﻿meaning that the factor was poorly defined and may not replicate well. The same issue appeared in ﻿the ECV for the specific factors. The ECV was strong for the general factor in the symmetrical bifactor model (ECV = 0.75) but ﻿weak for the specific factors, which indicates that the specific factors ﻿added little unique explanatory power. The ﻿ω_h_ was low for the general factor and specific factors for the symmetrical bifactor model, showing that the general factor ﻿accounted for ﻿little reliable variances. Therefore, for model validity and quality purposes, the bifactor (S–1) model was deemed more appropriate and accepted. The bifactor (S–1) model was also shown ﻿to be invariant across time (Table 7).

**Table 7:** Model invariance.

Models	*χ* ^2^	df	*p*	CFI	RMSEA	SRMR	AIC
Configural model	2016.88	840	<0.001	0.914	0.061	0.044	50872.91
Metric model	2059.46	883	<0.001	0.914	0.06	0.049	50829.49
Scalar model	2080.21	912	<0.001	0.915	0.059	0.049	50792.24

	|Δχ^2^|	df	*p*	ΔCFI	ΔRMSEA	ΔSRMR	ΔAIC
Metric against configural	42.58	43	0.49	0.000	0.001	0.005	43.42
Scalar against configural	63.33	72	0.76	0.001	0.001	0.005	80.67
Scalar against metric	20.75	29	0.87	0.001	0.001	0.000	37.25

### 3.3 Correlations between factor scores across models

**Figure 2:**
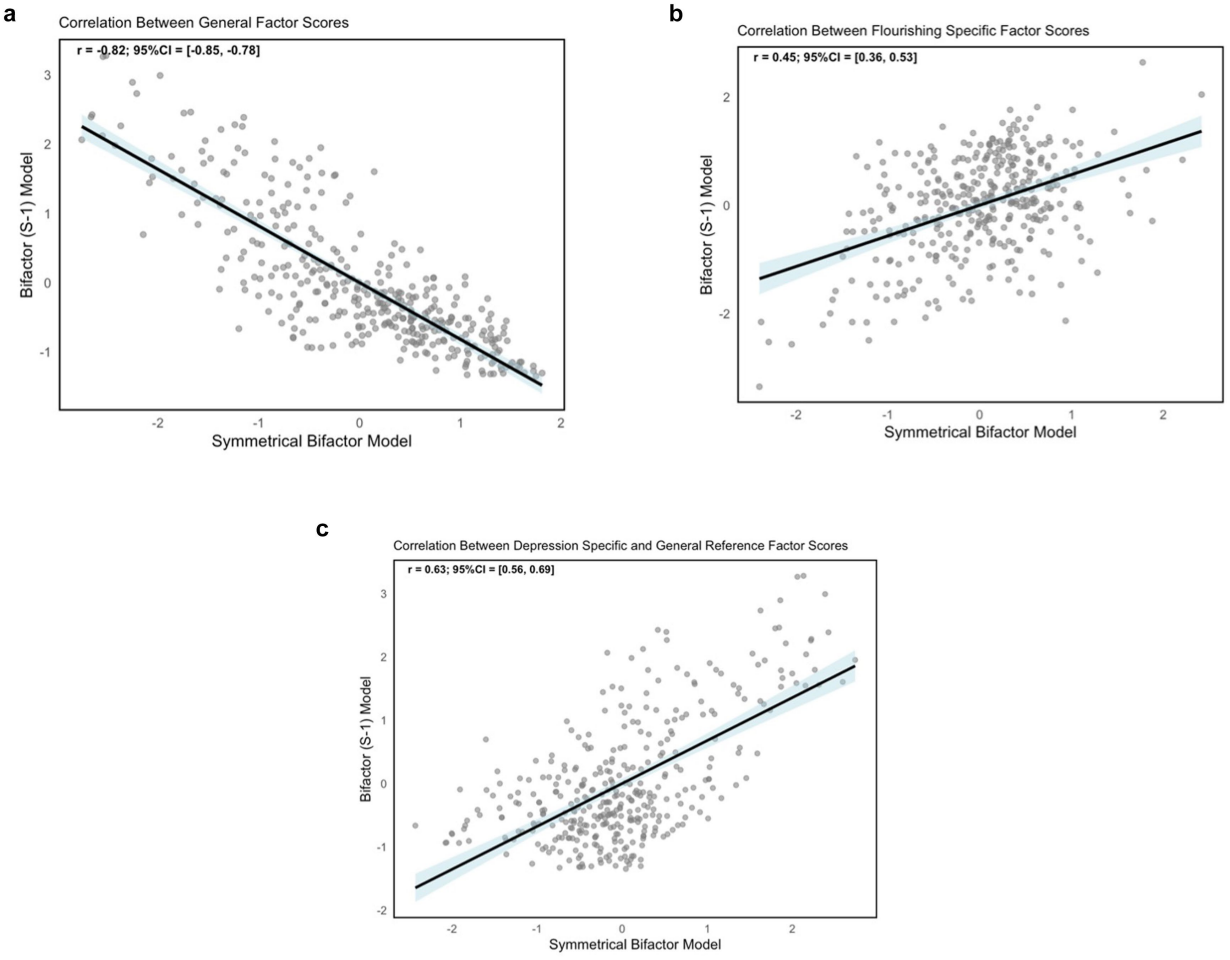
Correlations and scatterplots between estimated factor scores fitted in bifactor (S–1) (vertical axis) and symmetrical bifactor models (horizontal axis) **(a,b,c)**. Blue shared areas are the 95% confidence interval of the best-fit regression line.

As shown in Figures 2a,2b,2c, ﻿the estimated factor scores for the general factors between the symmetrical and (S–1) ﻿models were ﻿strongly correlated. In contrast, the specific flourishing﻿-specific factors across the symmetrical and (S–1) models were moderately correlated, possibly due to the anomalous loadings on the flourishing specific factor in the symmetrical bifactor model. Since the general reference factor from the bifactor (S–1) model was anchored by the depression items, it had a strong correlation with the depression﻿ specific factor from the symmetrical bifactor model. The correlation results reveal that the bifactor (S–1) model accommodates the essential features from the symmetrical bifactor model while enhancing the validity of the flourishing-specific factor. These correlations indicate structural convergence rather than construct equivalence.

### 3.4 Criterion validity

The SEM model had acceptable model fit indices ﻿of CFI = 0.92, TLI = 0.91, RMSEA = 0.04, and SRMR = 0.04. After controlling for age, gender, familial annual income, and baseline effects, the general reference and flourishing specific factor at T1 significantly predicted positive affect, engagement, relationship, meaning, and achievement ﻿constructs of the PERMA-Profiler at T2. Concurrently, the general reference factor at T1 was significantly associated with positive affect and meaning at T1. ﻿The﻿ flourishing specific factor was significantly ﻿correlated with positive affect, achievement, and meaning. ﻿The relationship construct was neither associated ﻿nor with the general reference factor nor flourishing specific factor. The standardized regression results are in Table 8.

**Table 8:** Standardized results of simultaneous regressions (within an SEM) of each ﻿criterion variable on the latent general reference factor and flourishing specific factor defined in a bifactor (S–1) model controlling for demographic covariates of no interest (age, sex, and annual familial income) and baseline effects.

﻿Factors and variables	Factors			
	General reference factor		Flourishing specific factor	
	*β* (SE)	*p*	*β* (SE)	*p*
Predictive				
Positive affect	0.33 (0.05)	**<0.001**	0.13 (0.04)	**0.004**
Engagement	0.23 (0.05)	**<0.001**	0.14 (0.05)	**0.003**
Relationship	0.15 (0.05)	**0.001**	0.13 (0.04)	**0.001**
Meaning	0.31 (0.05)	**<0.001**	0.20 (0.04)	**<0.001**
Achievement	0.27 (0.05)	**<0.001**	0.15 (0.04)	**<0.001**
Concurrent				
Positive affect	0.59 (0.06)	**<0.001**	0.17 (0.04)	**<0.001**
Engagement	0.17 (0.06)	**0.004**	0.13 (0.05)	**0.007**
Relationship	0.02 (0.12)	0.89	−0.01 (0.06)	0.91
Meaning	0.26 (0.04)	**<0.001**	0.12 (0.03)	**<0.001**
Achievement	0.66 (0.03)	**<0.001**	0.34 (0.04)	**<0.001**

Bolded are significant *p*-values. *p* < .01.

## 4 Discussion

To﻿ the best of our knowledge, this theory-driven study is the first to examine the longitudinal validity of the bifactor (S–1) model while controlling for confounding variables. Specifically, it demonstrates both longitudinal invariance and criterion validity when depressive symptoms are used as the reference facet. Considering the mixed evidence in the literature regarding whether the relationship between flourishing and depression is bipolar or bivariate, the bifactor (S–1) model offers a robust psychometric framework for understanding mental health. These findings underscore the importance of incorporating flourishing into intervention and prevention strategies, rather than focusing solely on symptom reduction. The applicability of the bifactor (S–1) model ﻿suggests its potential in modeling intervention changes and ﻿mental health.

The bifactor (S–1) model represents a psychometric variance structure that offers an alternative response to the bipolar/bivariate debate, although it does not resolve this theoretical issue. It differs from the correlated-factors model in that it does not primarily assume that the two concepts are separate and then test their relationship. Rather, it simultaneously treats flourishing and depressive symptoms as lying on the same continuum, consistent with recent psychometric evidence (Zhao and Tay, 2023, 2024), while also modeling a specific factor for flourishing. Thus, the model also supports the view that flourishing can vary independently from depressive symptoms, in line with the ﻿dual-factor model of mental health [Keyes (2002, 2006); Keyes et al. (2015); Keyes and Martin (2017)]. The specific flourishing﻿-specific factor in the bifactor (S–1) model has important practical implications, as it suggests that flourishing may be observed even among individuals who experience psychological symptoms. Although mental illness undeniably affects individuals﻿ who experience it, flourishing as a psychosocial outcome is also linked to meaning in life, social contribution, and social support from both the microsystem and mesosystem. Therefore, for a person with mental illness who has sufficient social support, flourishing remains possible. Prior research has identified﻿ subgroups who are “symptomatic but content” subgroups using latent profile analysis [Renshaw and Cohen (2014); Renshaw et al. (2016); Scutt et al. (2023); Suldo et al. (2011)]. Extending the ﻿existing literature background, the bifactor (S–1) model provides a framework that endorses the idea that people with mental illness can still thrive﻿. ﻿The framework can be applied to the design and evaluation of well-being interventions for individuals with chronic mental health conditions.

The psychometric indices holistically indicate that the bifactor (S–1) model provides a ﻿more robust and interpretable structure for the data compared ﻿with the symmetrical bifactor model. Both general and specific factors were measured reliably, and the model allows for nuanced understanding of multidimensional constructs by distinguishing between shared and unique variance. This supports the use of the bifactor (S–1) model for both research and applied assessment, especially when it is important to capture both general and domain-specific aspects of psychological functioning. The high ECV and H﻿-index values for the general factor indicate that a large ﻿proportion of the variance is shared across all items, supporting the presence of a strong general construct ﻿conprising depression plus the shared variance with flourishing. The specific factor also shows good reliability and a substantial ECV, indicating ﻿that it captures unique variance not explained by the general factor. This supports the theoretical importance of the specific domain (e.g., flourishing) within the broader construct. The high PUC and factor determinacy values suggest that the bifactor (S–1) model fits the data well and that the factor scores can be interpreted meaningfully. However, given the relatively low ﻿ω_h_ for the general reference factor, caution is warranted when interpreting total scores as representing only the general reference factor. Compared with the symmetrical bifactor model, the bifactor (S–1) model of mental health has better interpretability and psychometric strength, indicating better model validity and greater potential for application in future research.

The present study demonstrated the appropriateness of the bifactor (S–1) model through an exploratory analysis of attrition data, replication with a two-wave data, and a direct comparison with the symmetrical bifactor model. The initial analysis ﻿applied rigorous techniques, including EFA and EBFA, ﻿to the attrition sample independently of the longitudinal sample. The results supported the superiority of the bifactor model in capturing the dimensional structure of the constructs. These findings provided a foundation for assessing the model’s replicability. CFA conducted on the second-wave data of the longitudinal sample validated the initial results and demonstrated the model’s stability across time points. Model fit indices consistently favored the bifactor (S–1) structure, with lower ﻿χ^2^ values, and more favorable CFI and RMSEA statistics, indicating a more parsimonious and accurate representation of the data. Further comparison with the symmetrical bifactor model highlighted the limitations of the ﻿latter: specifically, its tendency to conflate specific factors with the general factor. For example, the regression coefficients between the flourishing-specific factor from the symmetrical bifactor model and the criterion variables were higher than those in the bifactor (S–1) model, while the ﻿ω_h_ and ECV of the flourishing-specific factor in the symmetrical bifactor model were low, indicating that the factor explained little variance. Therefore, the bifactor (S–1) model structure has shown more valid results when applied to psychosocial mechanism studies.

While Weijters et al. (2013) ﻿highlighted how formatting or text orientation can create artificial methods factors, depression and flourishing are distinct, substantive clinical and psychological constructs (Keyes, 2002)—not merely grammatically reversed equivalents of the same items. Therefore, the negative variance reflects true substantive covariance (ill-being vs. well-being) operating beneath an overarching general factor of psychological health. To explicitly test whether this pattern was an artifact of an unconstrained symmetrical model, we applied the bifactor (S–1) framework (Eid et al., 2017). By utilizing depressive ﻿symptoms as a well-defined reference factor, the bifactor (S–1) model successfully accommodated this bipolarity. The remaining specific factors retained stable, substantive meanings rather than collapsing into uninterpretable method residuals.

This study extends the application of the bifactor model of mental health to a Chinese sample, thereby broadening its relevance beyond Western contexts. Prior research on the bifactor or bifactor (S–1) model has primarily focused on samples from developed, Western countries, such as the United Kingdom (Black et al., 2019) and Australia (Hides et al., 2020). However, cultural influences on mental health perception and expression are well documented. For instance, Guo S. et al. (2015) found that Asian American adolescents were less likely than their White peers to seek help when facing psychological difficulties, highlighting cultural differences in mental health behaviors. Cross-cultural studies further suggest that the relationship between positive and negative aspects of mental health is not universal. Schönfeld et al. (2016) reported that the negative correlation between well-being and distress was weaker among Chinese participants ﻿than their German and Russian counterparts, indicating that cultural context shapes how mental states are experienced and reported. Similarly, Abdel-Khalek and Lester (2010) found a weaker association between happiness and depression in a Kuwaiti sample ﻿than in a U.S. sample. These findings underscore the importance of validating mental health models in non-WEIRD (Western, Educated, Industrialized, Rich, and Developed) populations. By demonstrating that the bifactor (S–1) model fits well within a Chinese sample, the present study contributes contextual Chinese evidence to the literature. It supports the model’s reliability while also promoting a more nuanced understanding of mental health that incorporates both universal patterns and culturally specific expressions.

This study is not without limitations. First, it relied exclusively on self-report data, meaning that the criterion variables were not measured independently from the target variables, which may introduce common method bias. To mitigate this, a repeated-measures design was employed to reduce potential temporal confounding. Nonetheless, future research should incorporate multiple data sources—such as informant reports, clinician ratings, or objective behavioral indicators—to enhance the validity of findings and address the inherent limitations of self-report methodologies. Second, while many studies utilize the MHC-SF as a single composite indicator of global flourishing (Xiao et al., 2021), its foundational validation studies [e.g., Lamers et al. (2011)] establish it as a three-factor structure encompassing emotional, psychological, and social well-being. To preserve model parsimony and focus on our bifactor (S–1) framework, we treated flourishing as a unidimensional counterpart to depression. However, this approach inadvertently masks the unique variances and potential differential relationships that specific well-being subscales might share with depressive symptoms. Future researchers should consider generalize the model using more diverse flourishing scales that can capture the multi-faceted sub-structure of flourishing without sacrificing model identification. Third, a notable limitation of the current study is our operationalization of depression as a unidimensional construct. Extant literature robustly demonstrates that depression is a multi-factorial and heterogeneous phenomenon encompassing distinct cognitive, affective, and somatic components. By evaluating depression as a single factor alongside flourishing within the bifactor (S–1) framework, our model may fail to capture unique variance and specific aspects inherent to these sub-dimensions. Future research should consider employing more granular, multi-factorial representations of psychological distress to better elucidate how specific depressive facets interact with flourishing. Another limitation of our split-sample approach is that despite demographic equivalence, the two samples differed significantly in baseline academic engagement and achievement. These disparities may reflect distinct sub-populations, necessitating caution when generalizing the structural findings across varied student strata. Future studies should replicate the bifactor (S–1) model using behaviorally homogenous cohorts to ensure that variations in path coefficients are not confounded by baseline performance differences.

## Data Availability

The raw data supporting the conclusions of this article will be made available by the authors, without undue reservation.

## Footnotes

1
https://www.credamo.com
