# Supplementary material for: Accommodating flourishing and depressive symptoms into the bifactor (S–1) model of mental health: longitudinal invariance and criterion validity
Source: Front Psychol. 2026 Aug 4;17:1752388. doi: 10.3389/fpsyg.2026.1752388 (PMC13481498; doi:10.3389/fpsyg.2026.1752388)
Supplement: Supplementary file 1 [file Table_1.DOCX]

Table S1

*Mean, Skewness, and Kurtosis of the Observed Variables*

|  | Mean | Std. Deviation | Skewness | Kurtosis |
| --- | --- | --- | --- | --- |
| F1.T1 | 4.43 | 0.80 | –0.60 | 1.22 |
| F1.T2 | 4.40 | 0.78 | –0.66 | 1.06 |
| F2.T1 | 4.45 | 1.04 | –0.54 | 0.41 |
| F2.T2 | 4.42 | 1.01 | –0.49 | 0.22 |
| F3.T1 | 4.06 | 1.10 | –0.68 | 0.39 |
| F3.T2 | 4.08 | 1.12 | –0.49 | 0.30 |
| F4.T1 | 3.18 | 1.27 | 0.21 | –0.48 |
| F4.T2 | 3.25 | 1.28 | 0.11 | –0.57 |
| F5.T1 | 4.10 | 1.37 | –0.42 | –0.63 |
| F5.T2 | 4.16 | 1.27 | –0.47 | –0.49 |
| F6.T1 | 3.94 | 1.36 | –0.34 | –0.68 |
| F6.T2 | 4.07 | 1.34 | –0.44 | –0.59 |
| F7.T1 | 3.50 | 1.45 | –0.22 | –0.91 |
| F7.T2 | 3.68 | 1.45 | –0.26 | –0.80 |
| F8.T1 | 4.05 | 1.37 | –0.58 | –0.31 |
| F8.T2 | 4.17 | 1.28 | –0.59 | –0.06 |
| F9.T1 | 4.27 | 1.26 | –0.67 | 0.05 |
| F9.T2 | 4.36 | 1.16 | –0.78 | 0.42 |
| F10.T1 | 4.54 | 1.03 | –0.62 | 0.45 |
| F10.T2 | 4.55 | 1.11 | –0.58 | –0.16 |
| F11.T1 | 4.23 | 1.18 | –0.56 | 0.12 |
| F11.T2 | 4.29 | 1.14 | –0.46 | –0.12 |
| F12.T1 | 4.01 | 1.24 | –0.31 | –0.52 |
| F12.T2 | 4.07 | 1.26 | –0.36 | –0.46 |
| F13.T1 | 3.94 | 1.37 | –0.36 | –0.54 |
| F13.T2 | 4.04 | 1.32 | –0.37 | –0.52 |
| F14.T1 | 3.85 | 1.40 | –0.18 | –0.89 |
| F14.T2 | 3.98 | 1.40 | –0.33 | –0.68 |
| D1.T1 | 1.36 | 0.76 | 0.37 | –0.13 |
| D1.T2 | 1.34 | 0.72 | 0.51 | 0.13 |
| D2.T1 | 0.71 | 0.79 | 0.90 | 0.17 |
| D2.T2 | 0.65 | 0.73 | 0.85 | 0.12 |
| D3.T1 | 0.74 | 0.84 | 0.90 | 0.05 |
| D3.T2 | 0.70 | 0.82 | 1.01 | 0.32 |
| D4.T1 | 0.81 | 0.78 | 0.72 | 0.04 |
| D4.T2 | 0.79 | 0.78 | 0.76 | 0.16 |
| D5.T1 | 1.06 | 0.82 | 0.49 | –0.19 |
| D5.T2 | 1.00 | 0.82 | 0.56 | –0.10 |
| D6.T1 | 0.97 | 0.81 | 0.58 | –0.07 |
| D6.T2 | 0.96 | 0.82 | 0.67 | 0.07 |
| D7.T1 | 0.78 | 0.81 | 0.67 | –0.46 |
| D7.T2 | 0.79 | 0.84 | 0.80 | –0.15 |
| D8.T1 | 1.09 | 0.89 | 0.39 | –0.66 |
| D8.T2 | 1.08 | 0.85 | 0.41 | –0.47 |
| D9.T1 | 0.69 | 0.83 | 0.85 | –0.37 |
| D9.T2 | 0.68 | 0.83 | 0.90 | –0.22 |
| D10.T1 | 0.52 | 0.72 | 1.22 | 0.83 |
| D10.T2 | 0.51 | 0.70 | 1.22 | 0.80 |
| D11.T1 | 0.98 | 0.81 | 0.62 | 0.08 |
| D11.T2 | 0.96 | 0.81 | 0.62 | 0.00 |
| D12.T1 | 1.09 | 0.80 | 0.33 | –0.39 |
| D12.T2 | 1.02 | 0.70 | 0.25 | –0.20 |
| D13.T1 | 0.77 | 0.75 | 0.76 | 0.29 |
| D13.T2 | 0.82 | 0.75 | 0.47 | –0.62 |
| D14.T1 | 0.73 | 0.85 | 0.94 | 0.06 |
| D14.T2 | 0.76 | 0.86 | 0.95 | 0.13 |
| D15.T1 | 0.55 | 0.77 | 1.40 | 1.50 |
| D15.T2 | 0.50 | 0.69 | 1.32 | 1.49 |
| D16.T1 | 1.03 | 0.87 | 0.46 | –0.52 |
| D16.T2 | 1.12 | 0.86 | 0.38 | –0.51 |
| D17.T1 | 0.44 | 0.66 | 1.38 | 1.36 |
| D17.T2 | 0.47 | 0.67 | 1.35 | 1.34 |
| D18.T1 | 1.08 | 0.83 | 0.36 | –0.46 |
| D18.T2 | 1.05 | 0.86 | 0.64 | –0.08 |
| D19.T1 | 0.47 | 0.74 | 1.60 | 2.13 |
| D19.T2 | 0.48 | 0.70 | 1.41 | 1.56 |
| D20.T1 | 0.42 | 0.65 | 1.50 | 2.00 |
| D20.T2 | 0.46 | 0.71 | 1.39 | 1.14 |
| PERMA.1.T1 | 7.49 | 1.31 | -1.02 | 1.95 |
| PERMA.1.T2 | 7.30 | 1.40 | –0.66 | 0.66 |
| PERMA.2.T1 | 7.55 | 1.64 | –0.84 | 0.73 |
| PERMA.2.T2 | 7.45 | 1.66 | -1.03 | 1.41 |
| PERMA.3.T1 | 7.51 | 1.66 | –0.81 | 0.86 |
| PERMA.3.T2 | 7.48 | 1.71 | –0.94 | 1.27 |
| PERMA.4.T1 | 7.59 | 1.69 | -1.14 | 2.18 |
| PERMA.4.T2 | 7.85 | 1.46 | –0.86 | 1.49 |
| PERMA.5.T1 | 7.79 | 1.74 | -1.10 | 1.70 |
| PERMA.5.T2 | 7.79 | 1.65 | –0.99 | 1.38 |
| PERMA.6.T1 | 7.84 | 1.76 | -1.23 | 1.93 |
| PERMA.6.T2 | 7.82 | 1.77 | -1.07 | 1.15 |
| PERMA.7.T1 | 7.36 | 1.94 | –0.95 | 0.61 |
| PERMA.7.T2 | 7.41 | 1.94 | –0.92 | 0.77 |
| PERMA.8.T1 | 7.27 | 1.90 | –0.71 | 0.26 |
| PERMA.8.T2 | 7.38 | 1.87 | –0.85 | 0.53 |
| PERMA.9.T1 | 7.37 | 1.94 | -1.02 | 0.70 |
| PERMA.9.T2 | 7.33 | 1.92 | -1.02 | 1.19 |
| PERMA.10.T1 | 6.99 | 1.80 | –0.82 | 0.62 |
| PERMA.10.T2 | 7.17 | 1.79 | –0.89 | 0.66 |
| PERMA.11.T1 | 7.22 | 1.89 | -1.01 | 0.74 |
| PERMA.11.T2 | 7.27 | 1.87 | -1.10 | 1.13 |
| PERMA.12.T1 | 7.29 | 1.88 | –0.85 | 0.54 |
| PERMA.12.T2 | 7.43 | 1.89 | -1.03 | 0.79 |
| PERMA.13.T1 | 7.52 | 1.69 | –0.96 | 0.83 |
| PERMA.13.T2 | 7.54 | 1.74 | -1.01 | 1.29 |
| PERMA.14.T1 | 7.35 | 1.72 | –0.90 | 0.91 |
| PERMA.14.T2 | 7.45 | 1.75 | –0.80 | 0.61 |
| PERMA.15.T1 | 7.09 | 2.10 | –0.80 | 0.13 |
| PERMA.15.T2 | 7.23 | 2.07 | –0.81 | 0.26 |
